# Phenotypic and Positron Emission Tomography with [18F]fluordeoxyglucose (FDG PET) differences in corticobasal syndrome: comparison of two cases

**DOI:** 10.1590/1980-5764-DN-2023-0085

**Published:** 2024-06-24

**Authors:** Thais Winkeler Beltrão, Eduardo Barbosa de Albuquerque Maranhão, Victor Adill Gomes Correia, Pedro Mota de Albuquerque, Mariana Gonçalves Maciel Pinheiro, Rayanne Acioli Lins Santos, Luiz Eduardo Duarte Borges Nunes, Simone Cristina Soares Brandão, Breno José Alencar Pires Barbosa

**Affiliations:** 1 Geriatrics Service, Hospital das Clínicas de Pernambuco, Empresa Brasileira de Serviços Hospitalares, Universidade Federal de Pernambuco, Recife PE, Brazil.; 2 Academic Area of Neuropsychiatry, Centro de Ciências Médicas, Universidade Federal de Pernambuco, Recife PE, Brazil.; 3 Afya Faculdade de Ciências Médicas de Jaboatão, Jaboatão dos Guararapes PE, Brazil.; 4 Nuclear Medicine Service, Hospital das Clínicas de Pernambuco, Empresa Brasileira de Serviços Hospitalares, Universidade Federal de Pernambuco, Recife PE, Brazil.; 5 Academic Area of Clinical Medicine, Centro de Ciências Médicas, Universidade Federal de Pernambuco, Recife PE, Brazil.

**Keywords:** Corticobasal Degeneration, Tauopathies, Alzheimer Disease, Positron-Emission Tomography, Degeneração Corticobasal, Tauopatias, Doença de Alzheimer, Tomografia por Emissão de Pósitrons

## Abstract

Corticobasal syndrome (CBS) is a rare cause of dementia and comprises varied combinations of subcortical signs (akinetic-rigid parkinsonism, dystonia, or myoclonus) with cortical signs (apraxia, alien hand or cortical sensory deficit), usually asymmetric. We aimed to report and compare the clinical and neuroimaging presentation of two patients diagnosed with CBS. While case 1 had severe non-fluent aphasia associated with mild apraxia and limb rigidity, case 2 had a more posterior cognitive impairment, with a different language pattern associated with marked visuospatial errors and hemineglect. FDG PET played a significant role in diagnosis, suggesting, in the first case, corticobasal degeneration and, in the second, Alzheimer's disease pattern. CBS has been widely studied with the advent of new *in vivo* methods such as brain FDG PET. Studies that deepen the phenotypic and biomarker heterogeneity of CBS will be of great importance for better classification, prognosis, and treatment of the condition.

## RESUMO

A síndrome corticobasal (SCB) é uma causa rara de demência e compreende combinações variadas de sinais subcorticais (parkinsonismo acinético-rígido, distonia ou mioclonias) com sinais corticais (apraxia, mão alienígena ou déficit sensorial cortical), geralmente assimétricos. Nosso objetivo foi relatar e comparar as apresentações clínica e de neuroimagem de dois pacientes com diagnóstico de SCB. Enquanto o caso 1 apresentava afasia grave não fluente associada a apraxia leve e rigidez de membros, o caso 2 exibia comprometimento cognitivo mais posterior, com padrão de linguagem distinto, erros visuoespaciais e heminegligência. O FDG PET teve papel significativo no diagnóstico, sugerindo, no primeiro caso, degeneração corticobasal e, no segundo, padrão Alzheimer. A SCB tem sido amplamente estudada com o advento de novos métodos *in vivo*, como o FDG PET cerebral. Estudos que aprofundem a heterogeneidade fenotípica e de biomarcadores da SCB serão de grande importância para melhor classificação, prognóstico e tratamento da doença.

## INTRODUCTION

Corticobasal Syndrome (CBS) is a neurodegenerative condition characterized by different combinations of motor and cognitive symptoms, leading to progressive neurological and functional deterioration. CBS is considered a rare cause of dementia and comprises varied combinations of subcortical signs (akinetic-rigid parkinsonism, dystonia, or myoclonus) with cortical signs (apraxia, alien hand, or cortical sensory deficit) and a notable asymmetric presentation^1^.

The term CBS concerns a purely phenotypic description and may be caused by different underlying neurodegenerative pathologies, including Corticobasal Degeneration (CBD), Alzheimer's Disease (AD), or Progressive Supranuclear Palsy (PSP)^2^. On the other hand, CBD refers to the pathological entity originally characterized by asymmetrical frontoparietal cortical atrophy, loss of neurons in the substantia nigra, and swelling of the neuronal cell bodies with achromatic cells^3^.

Given such heterogeneous presentations, the study of CBS becomes challenging in neurology and geriatrics clinics, where these patients are commonly examined in the context of dementia or Parkinsonian syndromes. Currently, in the absence of disease-modifying treatments, research in the area is extremely relevant. The volume of publications in this area is increasing, especially with a focus on clinical presentation, diagnostic criteria, and laboratory and imaging biomarkers such as Positron Emission Tomography with [18F]fluordeoxyglucose (FDG PET)^4^. This method, which assesses glucose metabolism, provides valuable information to support the diagnosis of neurodegenerative diseases in clinical and research settings^5,6^.

In the present study, we aimed to report and compare the neuroimaging presentation of two patients diagnosed with CBS.

## METHODS

This is a descriptive case report study. Demographic, anamnesis, medical evaluation, and neuroimaging data of two patients followed since 2022 at the specialized dementia outpatient at Hospital das Clínicas, Universidade Federal de Pernambuco (Recife, Brazil) were reviewed. The project was approved by the local Research Ethics Committee. The patients (or their legal representatives) signed an informed consent form.

## CASE REPORT

### Case 1

**Figure 1:**
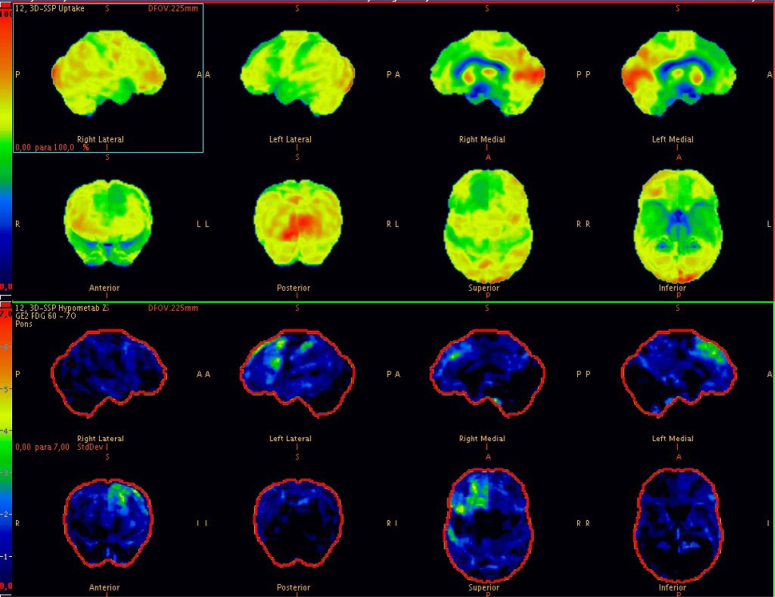
Metabolic map of brain Positron Emission Tomography with [18F]fluordeoxyglucose (FDG PET) showing asymmetric, left-predominant glycolytic hypometabolism in the middle frontal gyrus, anterior cingulate, accessory and supplementary motor cortex, as well as the dorsomedial prefrontal cortex, a pattern highly suggestive of a primary tauopathy.

The first patient was a 62-year-old female, with incomplete primary education and right-hand dominance. The condition began approximately two years before the first assessment, with difficulty finding words and communication impairments. She progressed over the following months with manual difficulties and motor limitations of the right upper limb. The limitations caused significant functional impairment: she could no longer cook, manage finances, or take medication. During the interview, she was alert and showed appropriate behavior, but in the language assessment there was a severe loss of fluency with pauses and hesitations. There was marked anomia and frequent use of generic terms (e.g. "thing, that"). She also performed occasional echolalia (e.g. repeating the last words of whatever she was asked). Sentence repetition, reading, and writing were also markedly impaired, but comprehension was relatively spared, as she could understand simple questions and commands. In the Mini-Mental State Examination (MMSE) she scored 16/30 and the Pfeffer Questionnaire was 20/30. She had bilateral ideomotor apraxia and bradykinesia more evident in the right upper limb with mild rigidity. Due to her language impairment, it was difficult to assess cortical sensory deficits such as agraphestesia or tactile agnosia. Dystonia, myoclonus, or other impairments were absent, and ocular movements showed preserved velocity in all directions. Laboratory screening for reversible causes of dementia was negative. Magnetic resonance imaging (MRI) of the skull showed cerebral atrophy with a slight predominance in the left perisylvian region. As this was an atypical dementia syndrome of early onset, an FDG PET was requested, which revealed asymmetric, left-predominant glycolytic hypometabolism in the middle frontal gyrus, anterior cingulate, accessory and supplementary motor cortex, as well as the dorsomedial prefrontal cortex, a pattern highly suggestive of a primary tauopathy (Figure 1). Due to the combination of cortical (aphasia, apraxia) and subcortical (rigid-akinetic parkinsonism) symptoms, the patient was diagnosed with possible CBS and is currently under medical and rehabilitation follow-up.

### Case 2

**Figure 2:**
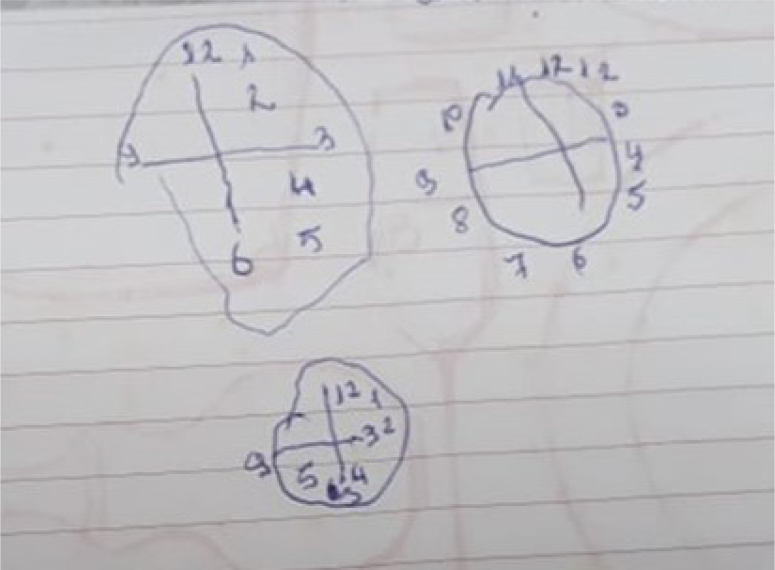
Clock Drawing Test in case 2, revealing serious difficulties in the spatial distribution of numbers and poor planning to perform the task.

**Figure 3:**
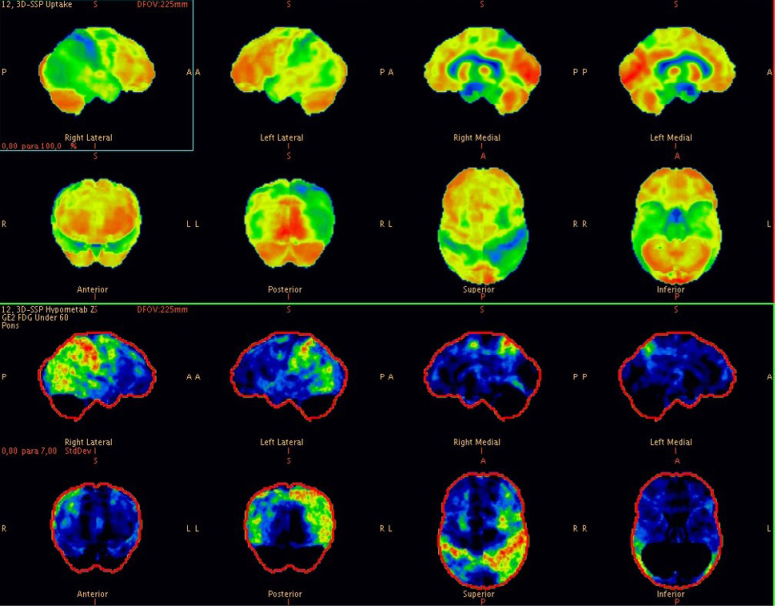
Metabolic map of brain Positron Emission Tomography with [18F]fluordeoxyglucose (FDG PET) study of case 2 revealing significant asymmetric, right-predominant glycolytic hypometabolism affecting posterior parietal regions and precuneus.

A 57-year-old female patient with a college degree education and right-hand dominance was referred for evaluation. Two years before her first consultation, she had been experiencing what she described as "feeling as if she unlearned how to use the car gear while driving" and difficulties getting dressed, situations accompanied by severe anxiety. She also described that her upper left limb seemed "forgotten". In the neurological evaluation, she was alert, attentive, and collaborative. She had evident bilateral ideomotor limb apraxia and non-fluent aphasia with pauses, hesitations, and circumlocutions. She also had left hemineglect characterized by sensory extinction and anosognosia. She scored 21/30 on the MMSE, and 18/30 on the Montreal Cognitive Assessment (MoCA) and showed significant visuospatial impairment on the Clock Drawing Test (Figure 2). Functional losses were moderate with a Pfeffer Score of 8/30. On somatic examination, we noticed bradykinesia, mild rigidity, and rare myoclonic jerks of the left hand. Laboratory screening for reversible causes of dementia was innocent and the brain MRI revealed disproportionate volumetric loss for age, more pronounced in biparietal regions, with mild asymmetry, worse on the left. Brain FDG PET showed significant asymmetric, right-predominant glycolytic hypometabolism affecting posterior parietal regions and precuneus (Figure 3). The patient was diagnosed with probable CBS. Given the early onset of the dementia syndrome, the patient and her family expressed interest in pursuing a study with AD biomarkers in the CSF, which revealed low levels of beta-amyloid, high levels of total tau and phosphotau, a pattern suggestive of CBS with evidence of AD pathology. There was a slight improvement in the following months with Donepezil 10 mg once a day and the patient is under regular follow-up with multidisciplinary rehabilitation.

## DISCUSSION

Both cases fulfill the criteria for CBS, but with different patterns of involvement: while case 1 had severe non-fluent aphasia associated with mild apraxia and limb rigidity, case 2 had a more posterior cognitive impairment, with a different language pattern associated with marked visuospatial errors and hemineglect (Table 1). As expected, FDG PET showed hypometabolism in different patterns: in the first case, a left frontal-predominant hypometabolism suggesting a CBD-pattern tauopathy and, in the second, an asymmetric posterior-predominant impairment suggestive of AD. This finding is in line with the literature, which points to the role of FDG PET in CBS: when due to AD pathology, hypometabolism predominates in the posterior temporoparietal areas and, when due to tau pathology, hypometabolism predominates in the thalamus and brainstem, mainly contralateral to the most affected side. Such contrasts were reinforced by an important study carried out by Parmera et al.^7^, who observed that the CBS group with PET FDG suggestive of AD showed worse cognitive performances, mainly in attention, memory, visuospatial domains, and presented more myoclonus in comparison to the non-AD group.

**Table 1:** Comparative synthesis between the clinical and imaging findings.

	Case 1	Case 2
Age, gender, education, onset of symptoms	62, female, 6 years of education 2 years of symptoms	57, female, college degree 2 years of symptoms
Cognitive evaluation	MMSE 16/30 Severe non-fluent aphasia Ideomotor apraxia	MMSE 21/30 Mild non-fluent aphasia Ideomotor apraxia Hemineglect
Somatic examination findings	Mild rigidity of the right upper limb	Bradykinesia Myoclonus Cortical sensory loss
Investigation	FDG PET: asymmetric, left-predominant glycolytic hypometabolism in the middle frontal gyrus, anterior cingulate, accessory and supplementary motor cortex, as well as the dorsomedial prefrontal cortex.	FDG PET: asymmetric, right-predominant glycolytic hypometabolism affecting posterior parietal regions and precuneus. Alzheimer's disease biomarkers: Amyloid-β (1-42): 497 ng/dL (normal >562) Phosphorylated tau: 130 ng/dL (normal <66) Total tau: 1,015.9 ng/dL (normal <370)

Abbreviation: MMSE, Mini-Mental State Examination.

The diagnostic classification of case 1 was particularly challenging due to its language-predominant presentation. The presence of one subcortical sign (asymmetric parkinsonism) with one cortical sign (limb apraxia) and non-fluent aphasia led us to classify the patient with possible (and not probable) CBS. While aphasia has not been included as a core cortical sign for the composition of the clinical criteria for CBS, language impairment has been considered frequent in this condition^8^. One study compared CBS-AD *vs*. CBS-non-AD discourse patterns and reported that the latter presented mainly with impairments related to motor speech disorders and syntactic complexity, as seen in the non-fluent variant of Primary Progressive Aphasia^9^.

Since PSP and CBS have significant phenotypic overlaps, another plausible possibility is that case 1 has a cortical variant of PSP, such as the corticobasal variant of PSP (PSP-CBS), a phenotype mostly linked to CBD pathology, although it might be found in cases with a PSP neuropathology^3^. Despite similar clinical manifestations between CBD and PSP, some clinical hints may favor CBD such as limb apraxia, asymmetrical bradykinesia, and increased saccadic latencies with preserved velocity in patients with CBD (as seen in our case). Finally, FDG PET can help the distinction between PSP and CBD. While FDG PET in PSP typically shows a characteristic pattern of hypometabolism in the midbrain, basal ganglia, thalamus, and frontal lobes, CBD asymmetrically involves the frontal and parietal lobes, basal ganglia, and thalamus^3^.

The study of the role of neuroimaging in CBS has increased in recent years. Although the most described pattern in CBS is asymmetrical atrophy of the parietal regions^3^, this finding may vary according to the underlying pathology. In a study carried out by Constantinides et al.^10^, MRI in patients classified as CBS due to CBD showed greater involvement of the premotor cortex, the supplementary motor area, and the insula. On the other hand, in CBS due to AD, there was greater involvement in temporal and posterior parietal regions. Perfusion studies of dopamine uptake in the basal ganglia do not discriminate very well between CBD and PSP, as low tracer uptake is seen in all conditions that co-occur with spontaneous parkinsonism, including Lewy Body Disease and Parkinson's Disease^11^.

Molecular neuroimaging using PET allows quantitative visualization of functional processes *in vivo*. FDG is the most commonly used radiopharmaceutical to assess regional brain glucose metabolism as a marker of neuronal function in neurodegenerative diseases^3^. In addition, pathology-specific ligands such as the amyloid-PET and tau markers are leading the way in neurodegenerative disease biomarkers with their role in revealing underlying pathology^5^. First-generation tau tracers such as flortaucipir showed good correspondence between *in vivo* imaging and postmortem PSP and CBD evaluation and second-generation tau PET tracers such as [18F]PI-2620 demonstrated more specificity for 4R-tauopathies^3,12^. In CBS, amyloid-PET can be a valuable tool to distinguish cases related to underlying AD pathology from cases related to CBD or PSP^3,13^.

CBS presents complex and distinct metabolic patterns due to its diverse pathologies. A recent study with neuropathological examination showed that the underlying pathologies of CBS are associated with different patterns of metabolic degeneration and hypometabolism described for CBS-CBD, CBS-AD, and CBS-PSP^14^. Another prospective study using FDG PET and amyloid-PET in a cohort showed that individual brain metabolic patterns could distinguish with high specificity and accuracy CBS due to AD from CBS due to non-AD variants, suggesting that it can be routinely used in clinical investigation from CBS^7^. In this study, the authors observed that individuals with CBS-AD showed hypometabolism in the posterior temporoparietal areas and precuneus, while individuals with CBS non-AD had a greater glycolytic reduction in the thalamus and brainstem, mainly contralateral to the most affected side, revealing possible metabolic signatures of CBS variants.

The present study is limited by its descriptive case report design, which does not allow inferring a robust association or causality between the findings described above. Another limitation is the unavailability of AD biomarkers in case 1. This is still the reality in most centers that care for patients with dementia^15^. Such biomarkers play an important role in the study of atypical forms of dementia, as in the cases described. Despite the limitations, the study of clinical cases continues to play a fundamental role in medicine due to its didactic potential. The reported cases present semiological and neuroimaging richness, with well-documented findings, which makes their description and comparison of great academic relevance.

In conclusion, CBS has been widely studied with the advent of new *in vivo* methods such as brain FDG PET. Studies that deepen the phenotypic and biomarker heterogeneity of CBS will be of great importance for better classification, prognosis, and treatment of the condition.
